# Disappearance of Melt Memory Effect with Comonomer Incorporation in Isodimorphic Random Copolyesters

**DOI:** 10.1021/acs.macromol.3c01389

**Published:** 2023-09-21

**Authors:** Leire Sangroniz, Maryam Safari, Antxon Martínez de Ilarduya, Haritz Sardon, Dario Cavallo, Alejandro J. Müller

**Affiliations:** † POLYMAT and Department of Polymers and Advanced Materials: Physics, Chemistry, and Technology, Faculty of Chemistry, University of the Basque Country UPV/EHU, Paseo Manuel de Lardizábal, 3, 20018 Donostia-San Sebastián, Spain; ‡ Physical Chemistry and Soft Matter, Wageningen University & Research, Wageningen 6708 WE, The Netherlands; § Department d’Enginyeria Química, Universitat Politècnica de Catalunya, ETSEIB, Diagonal 647, 08028 Barcelona, Spain; ∥ Department of Chemistry and Industrial Chemistry, University of Genova, Via Dodecaneso 31, 16146 Genova, Italy; ⊥ IKERBASQUE, Basque Foundation for Science, Plaza Euskadi 5, 48009 Bilbao, Spain

## Abstract

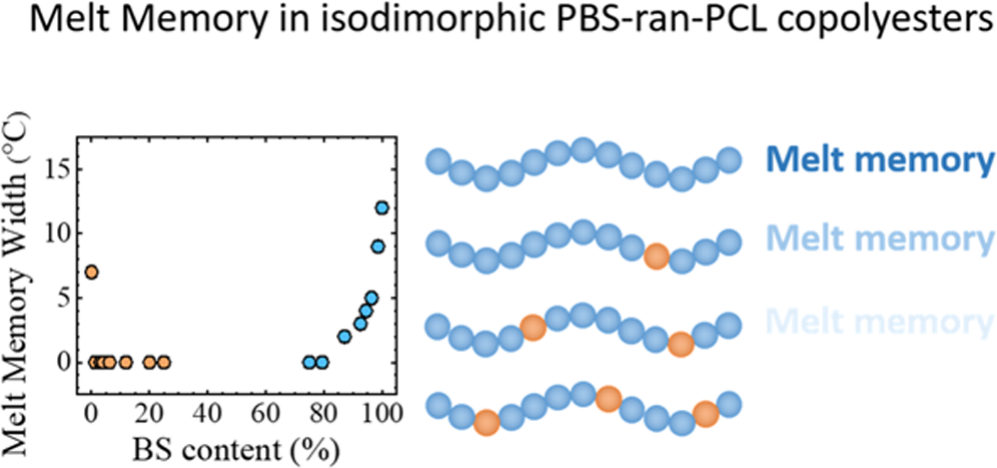

Melt memory effects in polymer crystallization have attracted much attention in the past few years. Although progress has been made in understanding how the chemical structure of polymers can affect melt memory, there are still some knowledge gaps. In this work, we study how incorporating a second comonomer unit that is partially included in the crystalline unit cell affects the melt memory effect of the major component in a random isodimorphic copolymer for the first time. This second comonomer unit depresses the melting temperature of the homopolymer, reduces the crystallinity, and distorts the crystalline unit cell. However, its effect on the stability of self-nuclei and the production of melt memory has not been studied so far. To this aim, we have selected poly[(butylene succinate)-*ran*-(ε-caprolactone)] random copolyesters PBS-*ran*-PCL that are isodimorphic, i.e., they exhibit a pseudoeutectic point. This point separates the formation of BS-rich crystals from CL-rich crystals as a function of composition. The results reveal that the melt memory effect of these isodimorphic copolymers is strongly reduced with the incorporation of even very small amounts of comonomer unit (i.e., 1 molar %). This indicates that the incorporation of a second comonomer unit in the polymer chain disrupts the intermolecular interactions present between the chain segments in the crystal lattice of the major component and reduces the capacity of the material to produce self-nuclei. This reduction is more drastic for copolymers in which the second comonomer unit is mostly rejected from the crystalline phase. Contrary to olefin-based copolymers, for copolyesters, the second comonomer unit eases the process to reach an isotropic melt state upon melting. This work reveals the impact of introducing comonomer units on the melt memory effect in isodimorphic random copolyesters.

## 1 Introduction

Polymers are widely used in various areas, including packaging, the automotive industry, construction, medicine, and everyday products like furniture or toys. The success of polymers results from their versatility, low density, easy processability, and low cost. However, the durability and high resistance to environmental factors have led to plastic waste pollution. Polymers are employed in single-used applications such as packaging, in which they are exploited only for a short period of time. Once they are disposed, they are incinerated, deposited in landfills, or recycled. However, some of the plastic waste escapes the collection and sorting process, leading to plastic waste accumulation in the environment.^1,2^

Due to the growing concern about plastic waste pollution, in the last decades, biodegradable polymers that can be degraded in bioactive environments have gained much attention. Under appropriate conditions, biodegradable polymers can be converted into carbon dioxide, methane, water, and biomass, among other substances.^3^ One of the most promising biodegradable polymers are polyesters, which can be obtained from renewable sources and are very versatile, with applications in several areas such as fibers, films, or bottles.^4^ Among biodegradable polyesters, polybutylene succinate (PBS) has interesting properties, such as good chemical resistance, biodegradability, and biocompatibility. It has a high melting temperature, above 100 °C, high heat deflection temperature, and can be obtained from renewable sources.^4−6^ However, due to the high crystallinity degree, it shows a brittle behavior as has been shown by some of us.^7^

One strategy to improve the physical performance of PBS in order to widen its possible applications is to copolymerize it with a second comonomer unit that has complementary properties. This is the case of polycaprolactone (PCL) that has interesting properties such as biocompatibility, good solubility, and excellent mechanical properties.^8^ In fact, PCL has a ductile behavior with elongation at break values^7^ above 100%.

The physical properties of poly[(butylene succinate)-*ran*-(ε-caprolactone)] copolymers PBS-*ran*-PCL have been intensely studied by some of the authors.^7−12^ The incorporation of the second comonomer units increases the ductility of the materials. On the contrary, the elastic modulus is reduced due to the lower crystallinity degree of the copolymers.^7^ The copolymers are able to crystallize for all compositions showing a reduction of melting and crystallization temperature with the incorporation of the second comonomer units.^9,10^ At a certain composition, two crystalline phases coexist, i.e., BS-rich and CL-rich phases. This composition is known as the pseudoeutectic point. The dimensions of the crystalline unit cell depend on the composition, showing variations with the presence of the second comonomer units. This behavior corresponds to isodimorphic copolymers in which the second comonomer units are partially included in the crystal lattice.^13^ The incorporation of the second comonomer units increases the nucleation rate and nucleation density of the material, although the spherulitic growth rate is reduced.^11^

It is well-known that the thermal properties of polymers depend on their thermal history. However, the effect of the temperature on the melt state has not been investigated for isodimorphic copolymers. When the thermal history of the polymer is completely removed by heating it to temperatures that are 25–30 °C higher than the melting temperature, an isotropic melt state is obtained. The polymer will crystallize at a standard crystallization temperature when it is cooled from this melt state. However, when the temperature is not enough to remove the thermal history, e.g., just above the melting temperature of the material, some self-nuclei can remain.^14,15^ In the subsequent cooling, the crystallization of the material will be shifted to higher temperatures than the standard value since those self-nuclei increase the nucleation density, which is proportional to the *T*_c_. When the polymer is heated to a temperature within the melting range, some crystals can survive that can act as self-seeds and could also anneal (if the temperature is low enough), this results in a rise in the crystallization temperature and in the following heating scan, part of the material could melt at higher temperatures (if annealing took place).

The mentioned self-nuclei have attracted much attention over the last years, and their exact nature is still under debate.^14,15^ According to differential scanning calorimetry (DSC) experiments, when the temperature is above the melting range of the polymer (i.e., above its end melting temperature, where no more endothermic heat is detected) but below the temperature needed to erase the thermal history, no crystal fragments remain. Other techniques, for instance, X-ray or optical microscopy, are not able to detect any differences between an isotropic melt state and melts containing self-nuclei. The effect of increasing the crystallization temperature produced by the generation of self-nuclei has been denoted the melt memory effect.^14,15^

Recently, the impact of the chemical structure of polymers on melt memory has been elucidated in a series of works covering polyamides, polyesters, polycarbonates, polyethers, or polyesters that contain several functional groups.^16−19^ These studies showed that melt memory is directly related to the strength of intermolecular interactions. The stronger the interactions, the more pronounced the melt memory effect, i.e., the increase of the crystallization temperature in comparison with the standard crystallization temperature will expand over a wider temperature range.^16,17,19^

The effect of incorporating a second comonomer unit on melt memory has been investigated in the case of polyolefins. Alamo et al. studied polyethylene-based copolymers.^20−24^ Their extensive studies show that the incorporation of a second comonomer unit, such as butene, results in strong melt memory effects that do not vanish even above the equilibrium melting temperature. This effect has been related to the formation of complex melt topologies due to the crystallizable sequence length selection during the crystallization process. Since the appropriate sequence has to diffuse to the crystal growth front, complex melt topologies are formed in the process. This impedes reaching an isotropic melt state, and thus, higher temperatures are required to reach this state in comparison with the homopolymer.^20−24^

Although there have been many previous studies on the melt memory effect, the impact of incorporating a second comonomer unit that can be partially incorporated within the crystalline unit cell of the homopolymer has never been investigated, as far as the authors are aware. The working hypothesis here is that taking into account that the comonomer units depress the melting temperature and modify the crystalline unit cell, it can modify the thermal stability of self-nuclei and thus alter the melt memory effect. It is relevant to study this effect since a stronger melt memory will require higher temperatures to remove the thermal history and thus a higher energy consumption during processing. In this work, we propose to investigate how small amounts of a second comonomer unit that can be partially incorporated within the crystalline unit cell of the homopolymer affect the melt memory effect in poly[(butylene succinate)-*ran*-(ε-caprolactone)] copolymers PBS-*ran*-PCL. This system has been selected because PBS and PCL homopolymers exhibit the widest melt memory effects among homopolymers,^15^ which allows to analyze precisely the impact of a second comonomer.

## 2 Experimental Part

### 2.1 Materials

**Figure 1:**
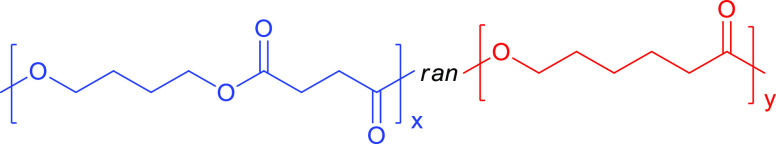
Chemical structure of PBS-*ran*-PCL copolymers.

PBS-*ran*-PCL copolymers rich in BS and CL were synthesized following the method described in a previous work.^10^ In brief, a two-stage melt polycondensation reaction was employed. First, a transesterification/ROP reaction was carried out with dimethyl succinate (DMS), 1,4-butanediol (BS), and ε-caprolactone (CL) at 160 °C under nitrogen for 4 h. After this step, polycondensation was conducted at 210 °C under reduced pressure for 4 h. The chemical structure of the copolymers obtained is shown in Figure 1. ^1^H NMR was performed to determine the composition and the number-average molar mass, see Table 1. Figure S1a,b shows ^1^H NMR of BS-phase and CL-phase, respectively. Based on our previous works with similar copolymers^10,11^ and the ^13^C NMR analysis carried out on selected compositions (Figure S2 and Table S1), the prepared copolymers can be considered random in the entire composition range. A comment about the molar mass is included in Supporting Information.

**Table 1:** Composition and Molar Mass of the Synthesized Copolymers

sample code	BS feed (% mol)	copolymer (% mol)	*M*_*n*_ (g mol^–1^)
BS_0_CL_100_	0	0.0	31,000
BS_1_CL_99_	1	1.3	12,100
BS_3_CL_97_	3	3.1	7950
BS_4_CL_96_	4	4.0	8880
BS_6_CL_94_	6	6.2	11,300
BS_12_CL_88_	10	11.8	18,100
BS_20_CL_80_	20	20.0	5900
BS_25_CL_75_	25	25.0	8700
BS_75_CL_25_	75	75.0	12,200
BS_80_CL_20_	80	79.4	15,350
BS_87_CL_13_	90	87.1	8980
BS_95_CL_5_	95	94.5	12,560
BS_97_CL3	97	96.4	14,000
BS_99_CL_1_	99	98.6	14,600
BS_100_CL_0_	100	100.0	32,700

### 2.2 Differential Scanning Calorimetry

The thermal properties were investigated employing a PerkinElmer 8500 DSC connected to an Intracooler III. The DSC was calibrated employing indium and tin, and the measurements were performed under ultrapure nitrogen flow.

#### 2.2.1 Nonisothermal Experiments

The material was heated to the appropriate temperature: 90 °C for CL-rich copolymers and 150 °C for BS-rich copolymers. The sample was held at this temperature for 3 min, and then, it was cooled to −60 °C. After holding the sample at −60 °C for 1 min, the sample was finally heated again. Heating and cooling rates of 20 °C min^–1^ were employed.

#### 2.2.2 Self-Nucleation Experiments

Melt memory effect was investigated applying the self-nucleation procedure developed by Fillon et al.^14,15,25^ The polymer sample was first heated to temperatures that are 25–30 °C above the end of the melting temperature to remove the thermal history. The sample was then cooled to −60 °C at 20 °C min^–1^, obtaining a standard crystalline state, and it was held for 1 min at −60 °C. Subsequently, it was heated to the selected *T*_s_ temperature and kept at this temperature for 5 min. After this step, the sample was cooled to −60 °C and then heated again at 20 °C min^–1^. With this procedure (Figure S3) and employing several *T*_s_ temperatures, the material can reach several *self-nucleation Domains*. Those are determined considering the crystallization temperature when cooling from *T*_s_ temperature and the melting temperature of a subsequent heating scan.^14,15,25^

### 2.3 X-ray Diffraction

The crystalline structure of the copolymers was analyzed by simultaneous wide-angle/small-angle X-ray scattering (WAXS/SAXS) at the ALBA Synchrotron radiation facility (Barcelona, Spain). The samples were prepared in DSC, removing the thermal history and creating a standard crystalline state. Then, the obtained DSC pans were measured in an X-ray facility. For the samples with a melting temperature close to room temperature, the standard crystalline state was created in situ in a Linkam THMS600 hot stage following the procedure employed in the DSC. In WAXS measurements, a Rayonix LX255-HS detector was employed (sample to detector distance = 99.25 mm, tilt angle = 30.04°). During SAXS experiments, a Pilatus 1 M detector was used (sample to detector distance = 6590 mm, tilt angle = 0°).

## 3 Results and Discussion

### 3.1 Nonisothermal DSC Experiments

**Figure 2:**
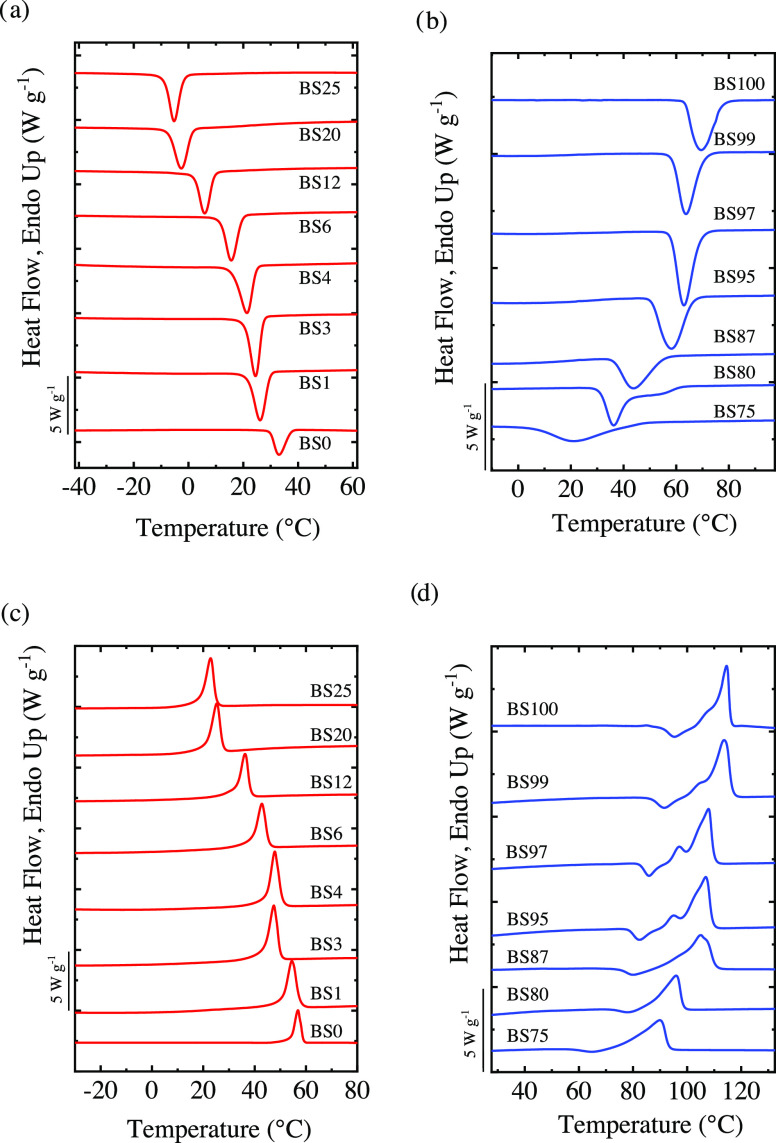
Cooling scans from the melt of (a) CL-rich and (b) BS-rich copolymers and subsequent heating scans of (c) CL-rich and (d) BS-rich copolymers at a rate of 20 °C min^–1^.

Figure 2 shows the cooling and subsequent heating scans obtained for PBS-*ran*-PCL copolymers rich in BS and CL. First, the thermal history was removed by heating the material to the appropriate temperature at 20 °C min^–1^. The thermal properties extracted from the heating/cooling scans of the homopolymers and copolymers are collected in Table S2. All of the copolymers rich in PCL or PBS can crystallize and show one crystallization peak, except BS_80_CL_20_, which shows two crystallization peaks. This copolymer only forms one crystalline phase, as it will be discussed in the next section (Crystalline Structure: X-ray Diffraction). For BS-rich copolymers, introducing CL comonomer units broadens the crystallization peak.

The subsequent heating scans (Figure 2c,d) show that PCL displays only one melting peak, whereas PBS shows an exotherm that arises from the recrystallization of imperfect/thinner crystals and a bimodal melting peak that probably corresponds to a melting recrystallization process during the heating scan.^10,26^ The copolymers show the same trend as that observed with the homopolymers: CL-rich copolymers show only one melting peak, whereas BS-rich copolymers show cold crystallization and bimodal melting peaks.^10^

**Figure 3:**
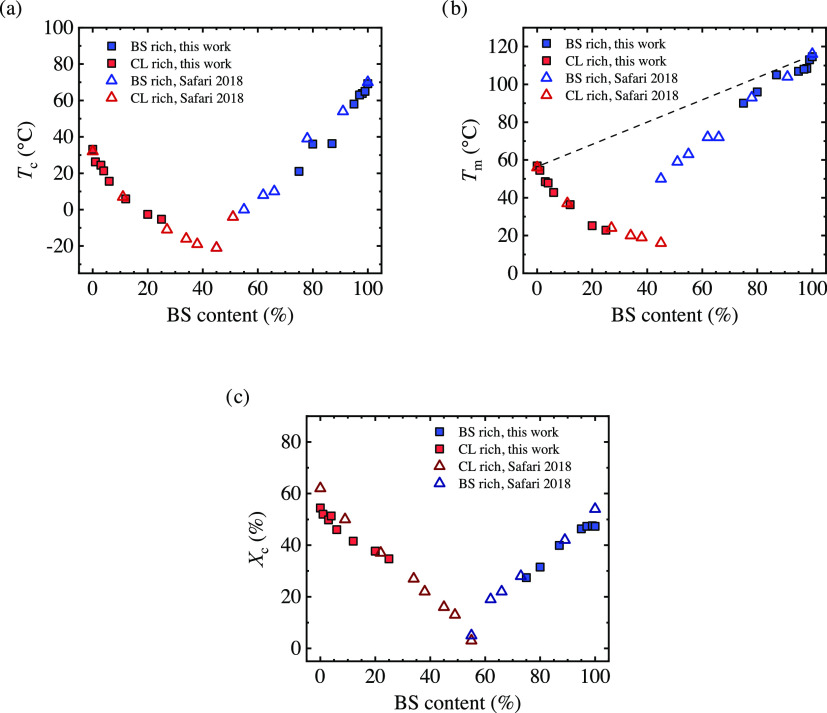
(a) Crystallization temperature, (b) melting temperature, and (c) crystallinity degree as a function of BS content. Data from Safari et al.^10^ has been included for comparison purposes.

Figure 3 shows the melting temperature (*T*_m_) and crystallization temperature (*T*_c_) as a function of BS content, including the results from previous studies covering the entire composition range. The results indicate that the copolymers crystallize under the conditions studied in this work in the whole composition range, with the *T*_m_ and *T*_c_ being a strong function of the composition of the comonomer units. In the plots of Figure 3, the characteristic pseudoeutectic behavior of isodimorphic random copolymers can be observed: a reduction of the transition temperatures with the incorporation of a second comononer unit reaching a minimum at an intermediate BS content.^10,13^ In random copolymers, *T*_m_ and *T*_c_ depend on the content and distribution of the comonomer units, with *T*_m_ being *T*_m_ controlled by lamellar thickness. The second comonomer units reduce the crystallizable chain length when it is excluded from the crystal lattice. Isodimorphic copolymers are capable of crystallizing in the whole composition range in unit cells that resemble the ones of the corresponding homopolymers with a small inclusion of the minor comonomer units. In these copolymers, an equilibrium exists between exclusion and inclusion of the comonomer units, where exclusion is usually more important.^13^ In fact, the reduction of the transition temperatures indicates that although some incorporation of the minor comonomer units occurs, the excluded amount of comonomer units from the crystalline phase is much higher than the inclusion, as has been proved for similar copolymers with X-ray and NMR techniques.^27^ However, the amount of inclusion depends on how different the chemical structures of the two monomers under consideration are. The more similar the chemical repeating units, the higher the inclusion amount. If the chemical structures are very similar, the copolymers can be isomorphic; in that case, total inclusion occurs.^13^

At the pseudoeutectic point, two crystalline phases coexist, as reflected by the two melting temperatures, corresponding to the BS- and CL-rich phases.^10,13^ Those crystalline phases can incorporate some units from the second comonomer. As shown in Figure 3, only one *T*_c_ is obtained at the pseudoeutectic composition, reflecting a coincident crystallization of both phases.

In Figure 3b, a line has been drawn by considering a simple law of mixtures regarding the melting temperature of homopolymers. This line corresponds to the complete inclusion of the comonomer units; therefore, the behavior indicated by this straight line corresponds to the trend displayed by hypothetical isomorphic copolymers.^13^ In the plot, we can observe that for CL-rich copolymers, there is a significant deviation from this line since there is a reduction in *T*_m_ instead of an increase with the BS content. This means that there is a significant exclusion of BS units from the CL crystalline phase. However, for BS-rich copolymers, the melting temperature does not deviate as much from the isomorphic line, which means that there should be a higher inclusion of CL comonomer units in the BS-rich crystals. As it has been reported in the literature, small comonomers units can accommodate more easily in the crystalline unit cell of bigger monomers, so this result could be expected.

Figure 3c shows the crystallinity degree calculated from the normalized melting enthalpy employing a value of 110.3 J g^–1^ as the equilibrium melting enthalpy^28^ (Δ*H*_m_^0^) for PBS and 139.5 J g^–1^ for PCL.^29^ A pseudoeutectic behavior similar to that observed for *T*_m_ is obtained with the composition of the copolymer. The reduction of *X*_c_ with the incorporation of the second comonomer units arises from the exclusion of the comonomer units that interrupt the crystallizable sequence segment.^10,13^

### 3.2 Crystalline Structure: X-ray Diffraction

**Figure 4:**
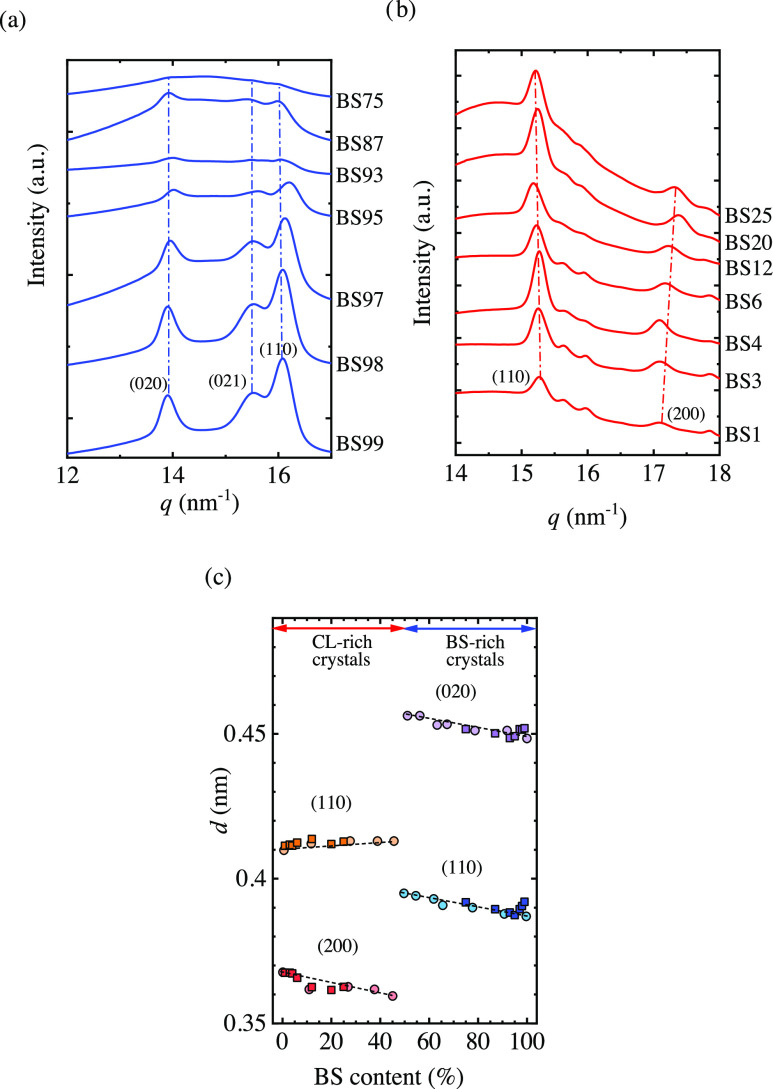
WAXS patterns for (a) BS-rich and (b) CL-rich copolymers. (c) *d*-Spacing for copolymers, including data from Safari et al.^10^ (circle symbols)

The crystalline structure of the copolymers was studied by WAXS. Figure 4 shows the WAXS patterns obtained at −50 °C after crystallizing the samples from the melt at 20 °C/min. The main reflections for BS-rich copolymers appear at 14, 15, and 16 nm^–1^ and correspond to the (020), (021), and (110) planes of the monoclinic unit cell of PBS. The results show that BS-rich copolymers form only BS-like crystals.^30,31^ Similarly, CL-rich copolymers show only CL-like crystals with primary reflections around 15.5 and 17.2 nm^–1^ that arise from the (110) and (200) planes of the orthorhombic unit cell^32−35^ of PCL. As has been reported previously by some of us,^9,10^ the PBS-*ran*-PCL copolymers at the pseudoeutectic point show reflections corresponding to both PCL and PBS crystals, confirming the existence of both crystalline phases.

The *d*-spacing from WAXS patterns has been calculated by employing Bragg′s law. The (110) and (020) planes of BS-like crystals and (100) of the CL-like crystals show changes in magnitude with comonomer content, which results from the modifications in the crystalline unit cell size (Figure 4c) needed for incorporation of comonomer units. These results indicate that some inclusion of comonomer units occurs in accordance with previous works on isodimorphic copolymers.^10,13^

**Figure 5:**
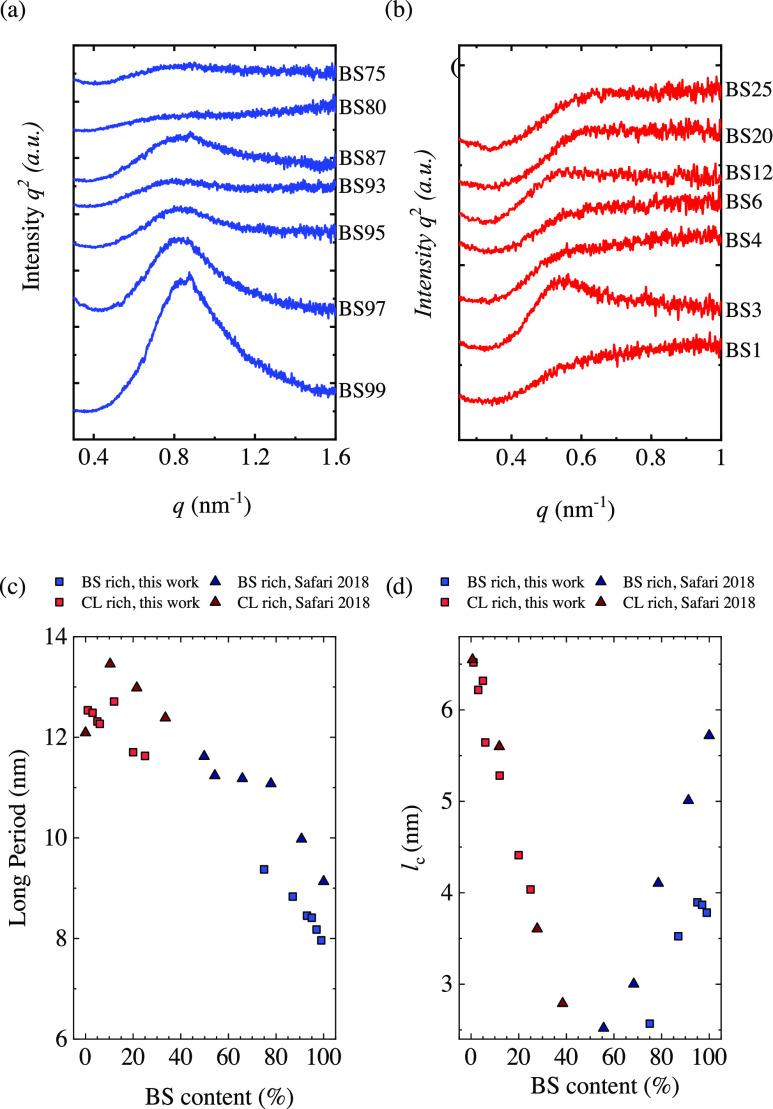
Lorentz-corrected SAXS profiles (*Iq*^2^ as a function of the scattering vector) for (a) BS-rich and (b) CL-rich copolymers. (c) Long period and (d) crystalline lamellar thickness for the copolymers, including the data covering the entire composition and reported before.^10^

SAXS experiments (Figure 5) were performed to determine the long period and lamellar thickness for the BS-rich and CL-rich copolymers. A single peak is observed for all of the copolymers, which arises from the X-ray scattering due to the lamella stacks in the sample. For some of the samples, the peak can not be observed (BS_80_), or the maximum is very weak; this could be due to the low crystallinity degree of the sample or to the fact that there are not enough lamella stacks fulfilling Bragg′s condition.

The long period can be obtained from the *q* value of the maxima of the peak. The long period as a function of composition is shown in Figure 5c. PBS has a long period close to 8 nm, whereas PCL shows a larger d* value of 12.5 nm. For BS-rich copolymers, the incorporation of CL increases the long period since PCL has a larger long period. Also, the presence of BS units in CL-rich copolymers increases slightly the long period. It should be considered that the long period depends on the crystallinity, so the results have to be considered cautiously.

Considering the long period and the crystallinity degree obtained by DSC, it is possible to estimate the crystalline lamellar thickness by employing the equation *l*_c_ = d*X_c_. In Figure 5d, the lamellar thickness as a function of copolymer composition is shown. The incorporation of the second comonomer units reduces the lamellar thickness. A pseudoeutectic behavior is observed similar to what has been obtained with melting and crystallization temperature or the crystallinity degree.^10,13^ The reduction of the lamellar thickness arises from excluding the comonomer units that determine the length of the crystallizable chain sequence.

### 3.3 Self-Nucleation Experiments

**Figure 6:**
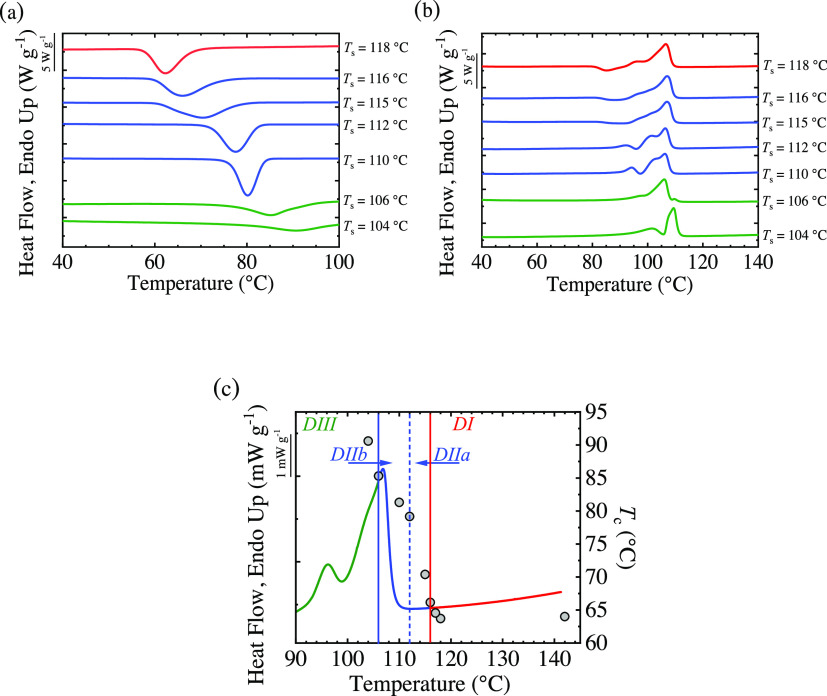
(a) DSC cooling curves obtained from several *T*_s_ values and (b) subsequent heating curves for BS_95_CL_5_ copolymer. (c) Standard heating endotherm superimposed to crystallization temperatures obtained at several *T*_s_ values. Vertical solid lines mark the transition between domains, whereas the dashed line indicates the end of the melting peak.

Figure 6 shows the DSC cooling curves from *T*_s_ temperature and posterior heating curves obtained at several self-nucleation temperatures for the BS_95_CL_5_ copolymer, as an example. When this copolymer is cooled from self-nucleation temperatures equal to or above 117 °C, it crystallizes at the same temperature. Thus, heating the material to 117 °C or above makes the material lose its thermal history and achieve an isotropic melt state. In the subsequent cooling, the material crystallizes at the standard crystallization temperature. So, at *T*_s_ equal to 117 °C or above, the polymer is in *Domain I* or the *isotropic melt Domain*.^14,15^

For *T*_s_ temperatures below 116 °C, the polymer crystallizes at higher temperatures during subsequent cooling compared to the standard *T*_c_ value. The increase in *T*_c_ is proportional to the increment in the nucleation density, which arises from the existence of self-nuclei. So, the 116–107 °C temperature range corresponds to the *self-nucleation Domain* or *Domain II*.

Finally, when *T*_s_ is low enough, some crystal fragments are left that are able to anneal. This occurs at temperatures equal to 106 °C or below. When cooling from the mentioned *T*_s_ region an increase in *T*_c_ is observed, and in the next heating scan, a second melting peak appears at higher temperatures due to the melting of annealed crystals. This additional melting peak marks the transition from *Domain II* to *Domain III*. This occurs at temperatures equal to 106 °C or below; at this temperature region, the material is in *Domain III* or the annealing and self-nucleation Domain.

The results obtained by applying the self-nucleation procedure are summarized in Figure 6c, in which a standard melting endotherm is superimposed with the crystallization temperatures obtained when cooling from several *T*_s_ temperatures. The vertical lines indicate the temperature range that comprises each *self-nucleation Domain*: At temperatures equal to or above 117 °C, the polymer is in *Domain I*; in the range 116–107 °C, the material is in *Domain II* and finally below 106 °C in *Domain III*.

Müller et al. proposed the split of *Domain II* into 2 sub-*Domains* considering the end of the melting temperature.^14,15,36^ The idea behind this division is to distinguish between the region in *Domain II* at high temperatures (i.e., *Domain IIa* or melt memory *Domain*), where according to the DSC data, all crystals are molten as no endothermic heat is detected, from that in *Domain II* at lower temperatures (*Domain IIb* or self-seeding *Domain*). In *Domain IIb* (or *DIIb*), the material is not fully molten, and there are some crystal fragments that behave as excellent self-seeds for nucleation.

For this BS_95_CL_5_ copolymer, the DSC final baseline after melting is reached at 112 °C, which means that, above this temperature, there are no crystal fragments that can be detected by DSC. Thus, in the 112–116 °C temperature region, the increment in the crystallization temperature comes from self-nuclei, and therefore, this region belongs to *Domain IIa* or to the melt memory *Domain*. Below 112 °C, DSC results indicate that there are some crystal fragments present since the baseline of the heat flow has not been reached. These crystal fragments are not able to anneal, and the sample is still considered to be in *Domain II*. So, this temperature region corresponds to *DIIb*, and the increase in *T*_c_ arises from the presence of self-seeds, and thus,*DIIb* is denoted by the self-seeding *Domain*.^14,15^

The results corresponding to all of the copolymers and homopolymers are displayed in the Supporting Information (Figure S4). The transition from *DII* to *DIII* occurs inside the melting endotherm for all of the copolymers, as could be expected. Regarding the transition from *DI* to *DII*, this is shifted to lower temperatures, getting closer to the melting temperature as the amount of the second comonomer units is increased. Thus, when a comonomer unit is incorporated, it is necessary to reduce the temperature more in comparison with the homopolymer to leave self-seeds that can efficiently increase the nucleation density, which results in an increase of *T*_c_. In other words, the incorporation of a comonomer unit facilitates the removal of the melt memory effect since lower superheatings are needed to obtain an isotropic melt state.

**Figure 7:**
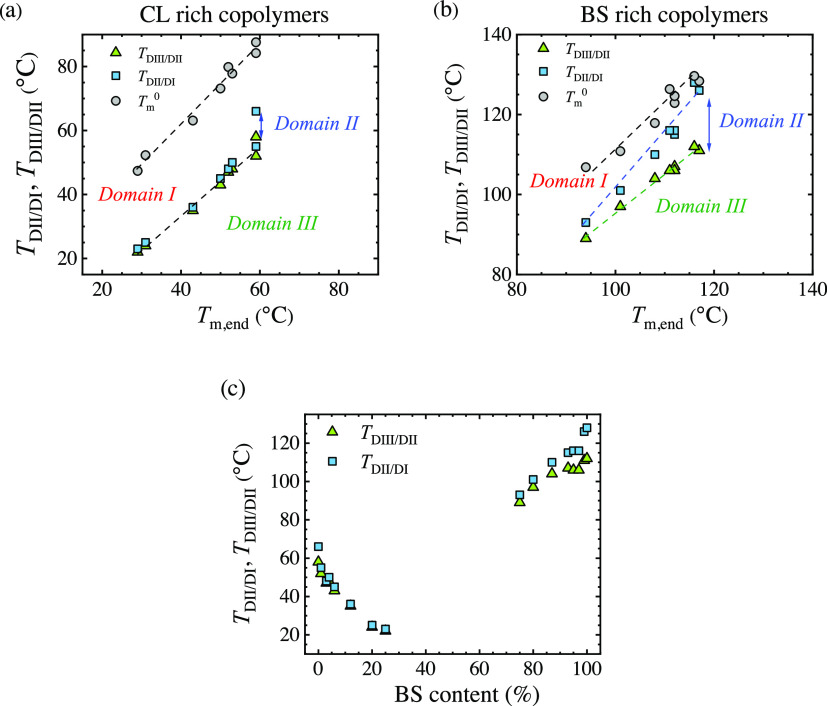
Transition temperatures between *Domain I*, *Domain II*, and *Domain III* and equilibrium melting temperatures (*T*_m_^0^) for (a) CL-rich copolymers and (b) BS-rich copolymers as a function of the end melting temperature. (c) Transition temperatures of the *Domains* as a function of BS content.

The transition temperatures that mark the limits of the self-nucleation domains are displayed as a function of the end melting temperature in Figure 7. The incorporation of a second comonomer unit reduces the melting temperature; therefore, in the plots of Figure 7a,b, the transition temperature between *Domains* of the neat homopolymer can be found at the highest *T*_m,end_ values of each plot. As the comonomer content increases, the transition temperatures corresponding to the copolymers progressively decrease as *T*_m,end_ values decrease.

For CL-rich copolymers, the temperature limits between *DII*/*DI* and *DIII*/*DII* lay very close to each other (Figure 7a), which reflects a narrow *Domain II*. Only the homopolymer and the copolymer with a small amount of comonomer units show some *Domain II*. There is a depression of the transition temperatures as *T*_m,end_ decreases or as the thermal stability of the standard crystalline state decreases, which could be expected considering that comonomer unit incorporation depresses the melting temperature. The slopes of both lines of transition temperatures versus *T*_m,end_ are close to one, reflecting a linear proportional behavior between the transition values and the thermal stability of the crystals.

On the contrary, for BS-rich copolymers (Figure 7b), a significant gap between the *DII*/*DI* and *DIII*/*DII* transition temperatures can be observed. Also in this case, the homopolymer and copolymers with a small amount of comonomer units show the most important *Domain II* width. The slope for the *DII*/*DI* transition is higher than 1 (1.1), whereas the one corresponding to *DIII*/*DII* is below 1 (0.94), which implies a faster depression for the melt memory effect with *T*_m,end_ decrease in comparison with the temperature required to anneal the crystals. For all copolymers, an isotropic melt state is reached well below the equilibrium melting temperature (*T*_m_^0^), as depicted in Figure 7a,b. The *T*_m_^0^ was taken from a previous work,^11^ and the values corresponding to the current compositions were estimated fitting the *T*_m_^0^ as a function of composition.

The results presented in Figure 7 indicate that the lower the melting temperature of the copolymers, the lower the increase in temperature to erase the melt memory effect is. A clear impact of the composition on the transition temperatures can be observed in Figure 7c, in which the transition temperatures are depicted along with the composition. The incorporation of a second comonomer unit reduces the transition temperatures for BS-rich and CL-rich copolymers.

**Figure 8:**
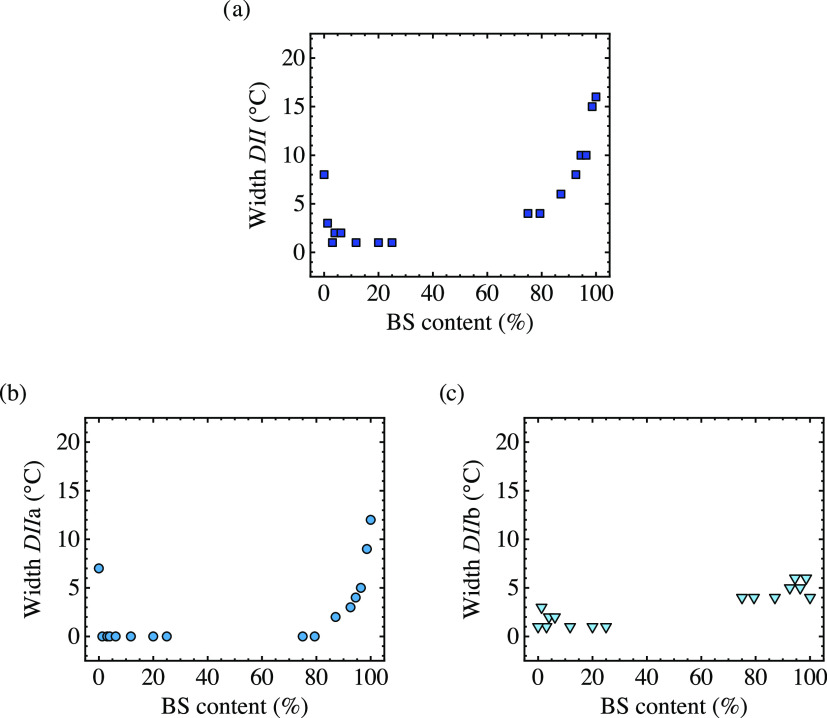
(a) *Domain II* width, (b) *Domain IIa* width, and (c) *Domain IIb* width as a function of BS content.

To consider the effect of the comonomer units incorporation on *Domain II*, Figure 8 shows *Domain II*, *Domain IIa*, and *Domain IIb* temperature widths as a function of copolymer composition. Incorporating a second comonomer unit reduces the width of *Domain II* progressively for copolymers rich in BS and CL.

If *Domain IIa* or the melt memory *Domain* is analyzed, the results are quite interesting (Figure 8b). PCL has a melt memory effect of 7 °C, which is close to the results reported in the literature.^37^ A slight incorporation of BS in PCL-rich copolymers (only 1.3%, according to Table 1) vanishes the entire melt memory effect. However, this is not the case for BS-rich copolymers. In this case, a progressive reduction in the width of *DIIa* is observed, and melt memory vanishes at a CL content of 20%. For most of the copolymers, there is some *DII* due to self-seeds, i.e., the magnitude of *DIIb* is always roughly constant (see Figure 8c). This means that there is only an increase of *T*_c_ in comparison with the standard *T*_c_ when the polymer is heated close to the end of the melting enotherm, leaving some small crystal fragments that cannot anneal.^15^

As mentioned previously, the melt memory of CL-rich copolymers is reduced with the incorporation of tiny amounts of BS (1.3%) comonomer units. However, in the case of BS-rich copolymers, higher amounts of CL (20%) are needed to erase the melt memory. A possible explanation for these differences lies in the exclusion/inclusion ratio of the second comonomer units. As mentioned before, considering the melting temperature behavior, there is a significant exclusion of BS units from the CL-rich crystalline phase, whereas CL units are included in a higher amount within the BS-rich crystalline phase. This is probably due to the larger size of BS monomers in comparison with CL monomers (see Figure 1).

The results indicate that the inclusion of the CL monomer in BS-rich crystals allows holding some melt memory effect, at least until 20% of CL incorporation. On the contrary, for CL-rich copolymers, BS is mainly rejected from the crystalline phase, and in this case, even a small presence of BS in the copolymer is enough to erase the melt memory effect. Thus, BS units reduce the thermal stability of self-nuclei and act as diluents, reducing the temperature needed to reach an isotropic melt state.

The results can also be explained by considering the intermolecular interactions. PBS shows a stronger melt memory effect that may arise from stronger interactions in comparison with PCL. The studies carried out with Raman spectroscopy, infrared spectroscopy, and quantum chemical calculations (QCCs) show that PBS and PCL have 3 types of hydrogen bonds comprising intermolecular and intramolecular interactions.^38,39^ Those hydrogen bonds are formed between the oxygen atoms from the ester groups and hydrogen atoms from methylene groups for PBS and PCL. These bonds have a similar bond length. However, it should be considered that for PCL, there are 5 methylene groups per each ester group, whereas for PBS, there are 6 methylene groups per 2 ester groups. This could explain why PBS shows a stronger melt memory effect than PCL since the intermolecular interactions are more diluted for PCL as it contains more methylene groups per functional group compared with PBS.

For the PBS-rich compositions, when the second comonomer units are introduced in the polymer chain and partially included in the crystal, these counits disrupt the mentioned interactions, which results in a reduction of the melt memory effect. In the case of PCL-rich compositions, the BS counits are largely excluded from the crystal, as judged by the melting point decrease. Nevertheless, the incorporation of only 1 mol % completely erased the melt memory. This unexpected result suggests that BS units, even if mostly rejected from the crystals, could still be located at the crystal surface, weakening the intermolecular interactions between CL segments. Schäfer et al.^27^ demonstrated by solid-state NMR studies that in PBS-*ran*-PBA isodimorphic copolymers, up to 9% PBS could be included within the crystal lattice of PBA in a 20:80 PBS-*ran*-PBA random copolymer. However, in the case of a 60:40 PBS-*ran*-PBA random copolymer, PBA repeating units were located on the crystalline surface, presumably in pocket-like structures (approximately 7 to 9% of BA units).

Despite the quantitative differences observed on the two sides of the composition diagram, the new and important finding is the reduction of the melt memory effect with small amounts of comonomer unit incorporation, which holds for both types of crystals and is thus related to an interplay between intermolecular interactions and comonomer unit inclusion/exclusion balance in the crystalline lattice.

This behavior differs completely from that of polyolefin-based copolymers in which the second comonomer units induce complex melt topologies, leading to a strong melt memory effect.^20−24,40^ Extensive works with ethylene-based copolymers have shown that incorporation of butene comonomer units^20,23,24^ results in a strong melt memory effect that persists even above the calculated equilibrium melting temperature. This melt memory originates from the sequence partitioning that occurs during the crystallization process. The comonomer units are excluded from the crystalline phase, and during crystallization, there is a selection of the sequence length; first, the longer sequences are selected, which have to diffuse to the crystal front. This process leads to a complex melt topology, resulting in a strong melt memory effect since higher temperatures are required to reach an isotropic melt state starting from the “heterogeneous” melt.^20−24^

For propylene/ethylene random copolymers, it has also been shown that the incorporation of ethylene comonomer units widens the melt memory effect, i.e., the melt memory effect is displayed on a broader temperature range.^39^ The results are explained considering the topological constraints brought by incorporating a second comonomer unit partially rejected from the crystalline phase, as for PE-based copolymers. However, for polypropylene-based copolymers, melt memory disappears at temperatures much lower than the equilibrium melting temperature.

The opposite trends between olefin-based copolymers and polyester-based copolymers could arise from the fact that the mechanisms for melt memory are different. In olefin-based copolymers, there are only weak van der Waals interactions. The melt memory effect in those materials arises from the complex melt topology that is generated due to sequence selection during crystallization and diffusion of the chain segments to the crystal growth front. However, in polyester and several polymers that contain functional groups such as polyamides, polyethers, and polycarbonates, melt memory is directly related to the intermolecular interaction strength. The stronger the interactions are, the stronger the melt memory effect.^16−19^ The incorporation of a second comonomer unit might reduce or disrupt the intermolecular interactions within the crystals on both sides of the pseudoeutectic point.

## 4 Conclusions

In this work, the melt memory effect of PBS-*ran*-PCL isodimorphic copolyesters was investigated for the first time. The copolymers with small amounts of a second comonomer unit show only one crystalline phase, similar to the one of the homopolymer. The study reveals that the incorporation of a second comonomer units strongly reduces the melt memory effect due to a disruption of the intermolecular interactions between the chain segments, easing the process to reach the isotropic melt state. The comonomer with a higher exclusion ratio, butylene succinate, shows a more severe reduction in the melt memory effect of polycaprolactone, which reflects a higher effectiveness in reducing intermolecular interactions within CL chain segments. This behavior is completely different from the behavior shown by olefin-based copolymers, which suggests that the mechanism of melt memory is different for those materials. In the case of polyesters, melt memory arises from intermolecular interactions present in polyesters. The second comonomer units dilute these interactions and result in melt topologies upon crystallization that are less segregated than those of olefin-based copolymers.

## Supporting Information Available

^1^H NMR of all the copolymers and ^13^C NMR of selected copolymers; table with the randomness of selected copolymers; the thermal SN procedure employed; the thermal properties obtained by nonisothermal DSC experiments and SN results of all the copolymers and homopolymers (PDF)
