# Supplementary material for: Disappearance of Melt Memory Effect with Comonomer Incorporation in Isodimorphic Random Copolyesters
Source: Macromolecules. 2023 Sep 21;56(19):7879–88. doi: 10.1021/acs.macromol.3c01389 (PMC10569436; doi:10.1021/acs.macromol.3c01389)
Supplement: Supplementary file 1 — ma3c01389_si_001.pdf [file ma3c01389_si_001.pdf]

Supporting Information

Disappearance of melt memory effect with comonomer incorporation in isodimorphic random copolyesters

Leire Sangroniz[1*], Maryam Safari[1,2], Antxon Martínez de Ilarduya[3], Haritz Sardon[1], Dario Cavallo[4], Alejandro J. Müller[1,5*]

[1]POLYMAT and Department of Polymers and Advanced Materials: Physics, Chemistry and Technology, Faculty of Chemistry, University of the Basque Country UPV/EHU, Paseo Manuel de Lardizábal, 3, 20018 Donostia-San Sebastián, Spain.

[2]Physical Chemistry and Soft Matter, Wageningen University & Research, Wageningen 6708 WE, the Netherlands

[3]Department d'Enginyeria Química, Universitat Politècnica de Catalunya, ETSEIB, Diagonal 647, 08028 Barcelona, Spain

[4]Department of Chemistry and Industrial Chemistry, University of Genova, Via Dodecaneso 31, 16146, Genova, Italy

[5]IKERBASQUE, Basque Foundation for Science, Plaza Euskadi 5, 48009, Bilbao, Spain.

(a) ^{1}H NMR of BS rich copolymers

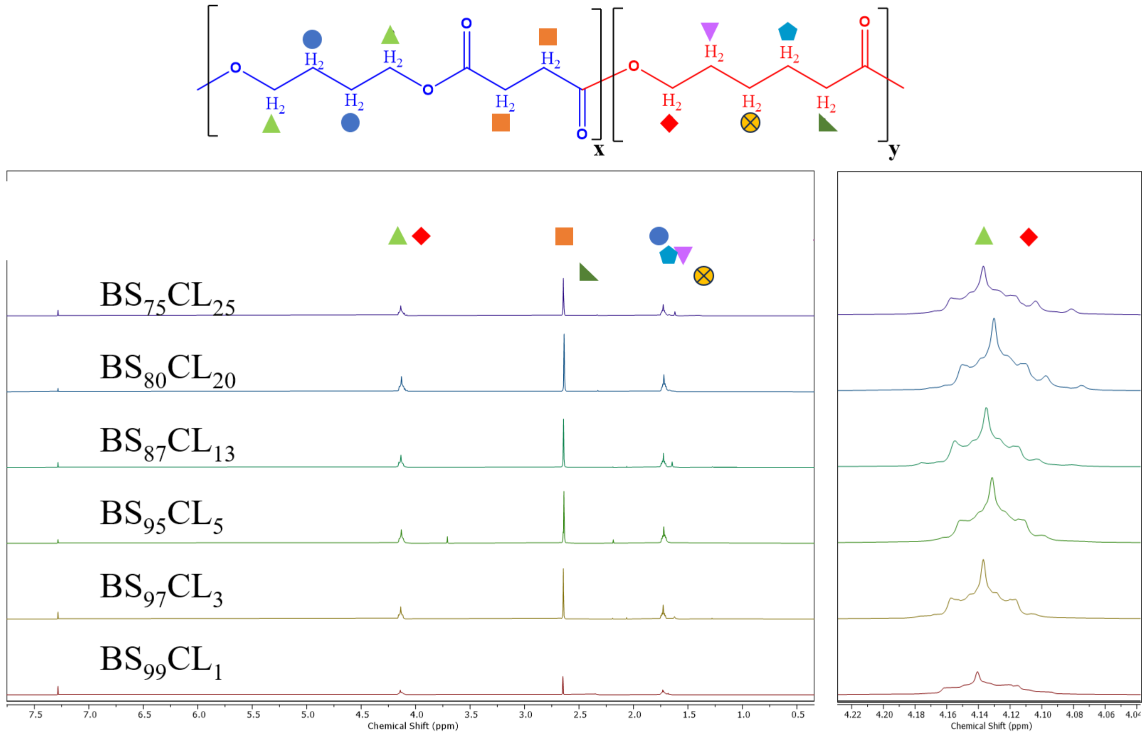

(b) ^{1}H NMR of CL rich copolymers

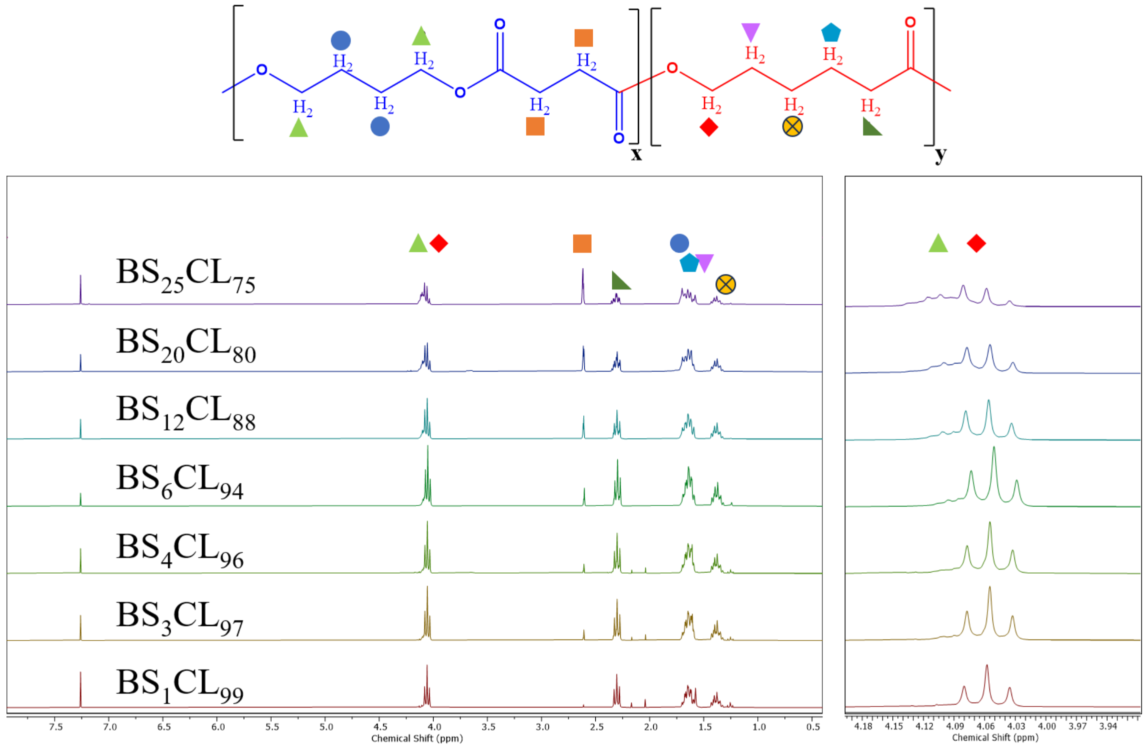

Figure S1. ^{1}H NMR spectra of BS rich and CL rich copolymers.

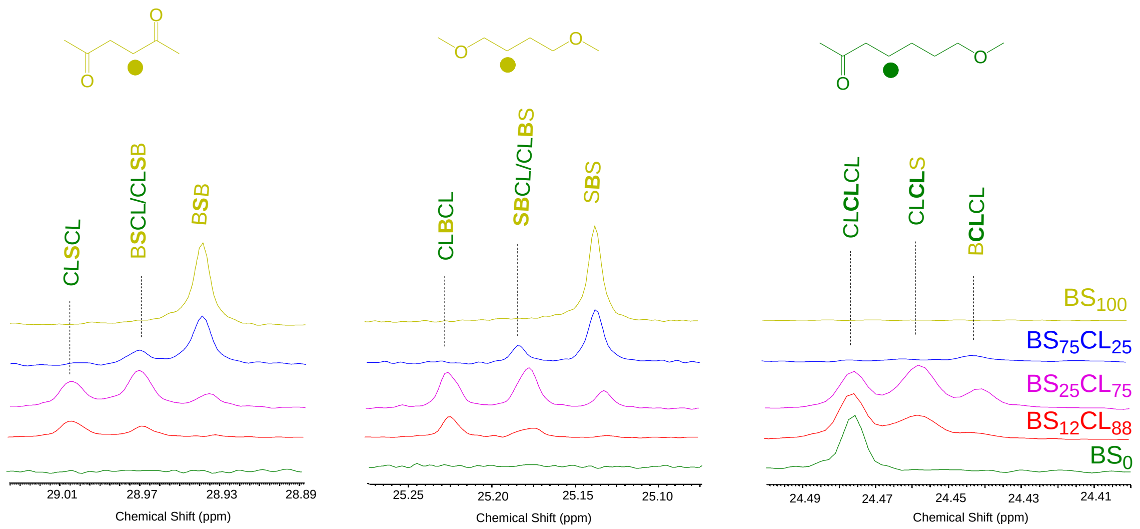

Figure S2. ^{13}C NMR spectra of selected copolymers and the homopolymers.

Table 1. Microstructure of BS_xCL_y copolyesters

Copolyesters	Microstructure [1]			
	CL**B**CL	CL**BS**/S**BC**L	SBS	***R***
$BS_{75}CL_{25}$	0	23.0	77.0	0.95
$BS_{25}CL_{75}$	38.1	45.9	16.1	0.99
$BS_{12}CL_{88}$	65.9	34.9	3.2	1.04

[1] Microstructure of copolyesters determined by ^{13}C-NMR. Signals from inner methylenes of butylene units that appear at 25.2 ppm were used for quantification of different triads centered in these units. *R*: degree of randomness calculated using equations described in reference [1].

Comment on Molar Mass

The homopolymers show slightly higher molar mass than the copolymers. However, recent studies with polycaprolactone show that for PCL with molar mass above 10 kg/mol there are no significant differences in melt memory [2]. So, the comparison of the homopolymers and copolymers is appropriate.

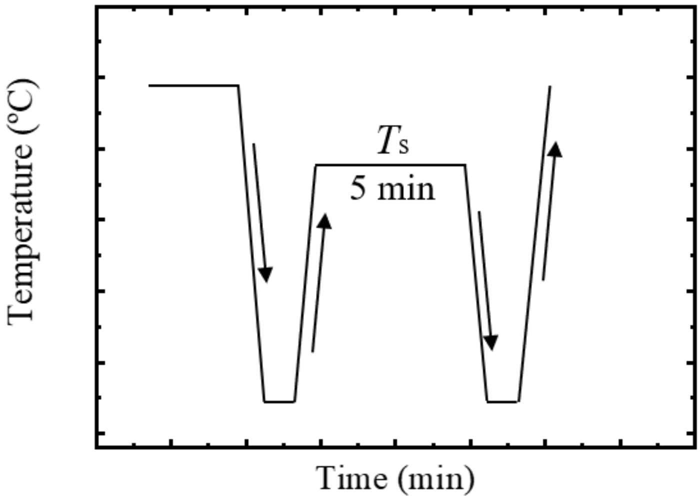

Figure S3. Schematic representation of self-nucleation thermal procedure.

Table S2. Thermal properties of the homopolymers and the copolymers.

	Sample code	T_c (ºC)	ΔH_c (J·g-1)	T_m (ºC)	ΔH_m (J·g-1)	X_c (%)
1	BS0	33.1	60	56.8	60	54.4
2	BS_1CL_{99}	26.2	58	54.5	58	52.0
3	BS_3CL_{97}	24.4	59	48.5	61	49.8
4	BS_4CL_{96}	21.3	59	47.8	59	51.3
5	BS_6CL_{94}	15.6	52	42.8	54	46.0
6	$BS_{12}CL_{88}$	5.9	49	36.3	52	41.55
7	$BS_{20}CL_{80}$	-2.6	51	25.2	52	37.7
8	$BS_{25}CL_{75}$	-5.3	50	22.8	51	34.7
10	$BS_{75}CL_{25}$	21.0	50	90.0	51	27.4
11	$BS_{80}CL_{20}$	36.0	53	96.0	55	31.5
12	$BS_{87}CL_{13}$	36.3	54	105.0	64	39.9

13	$BS_{95}CL_5$	58.0	65	106.9	68	46.3
14	$BS_{97}CL_3$	63.0	66	108.0	68	47.3
15	$BS_{99}CL_1$	65.0	62	113.0	67	47.5
16	BS_{100}	69.2	59	114.6	66	47.3

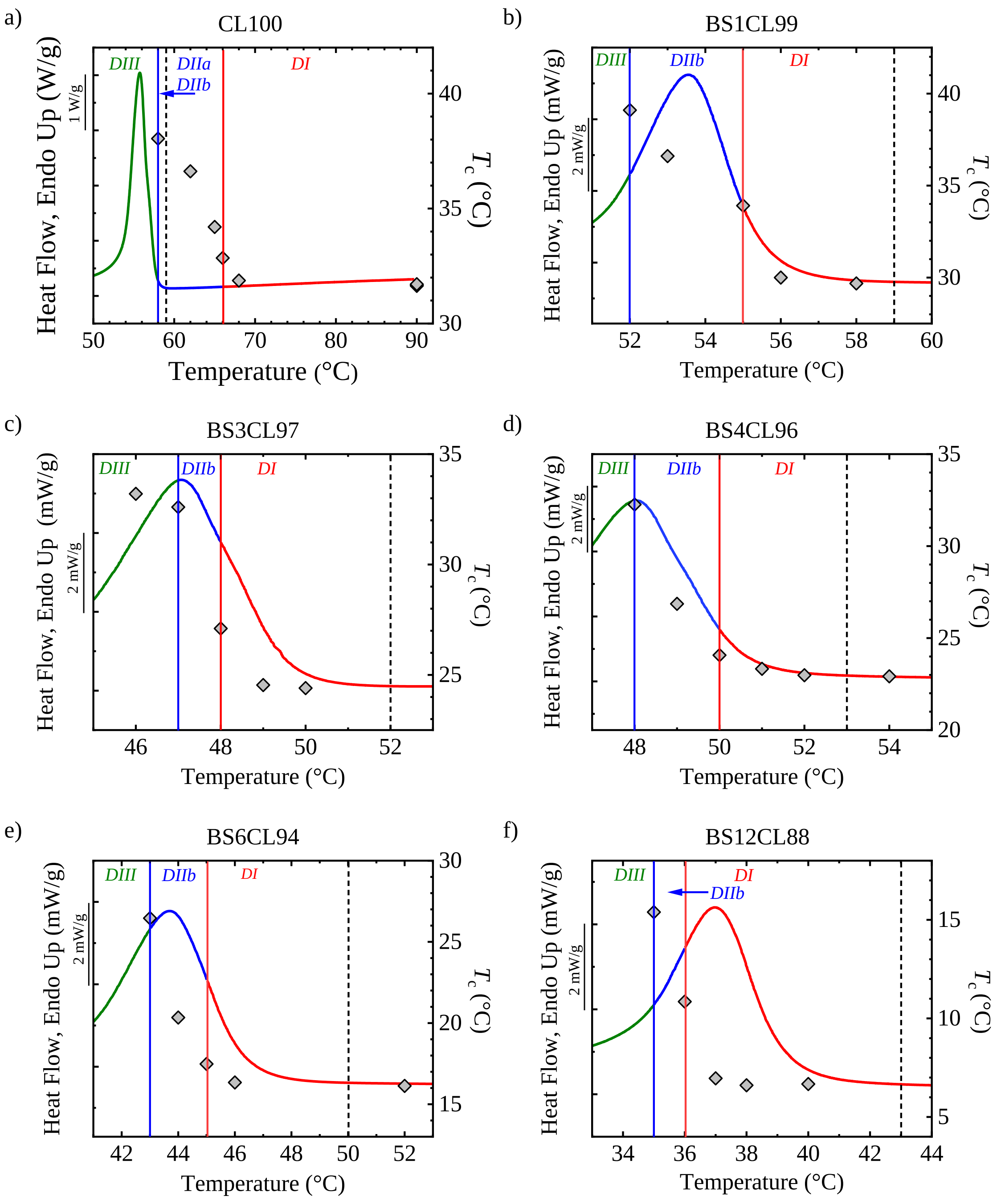

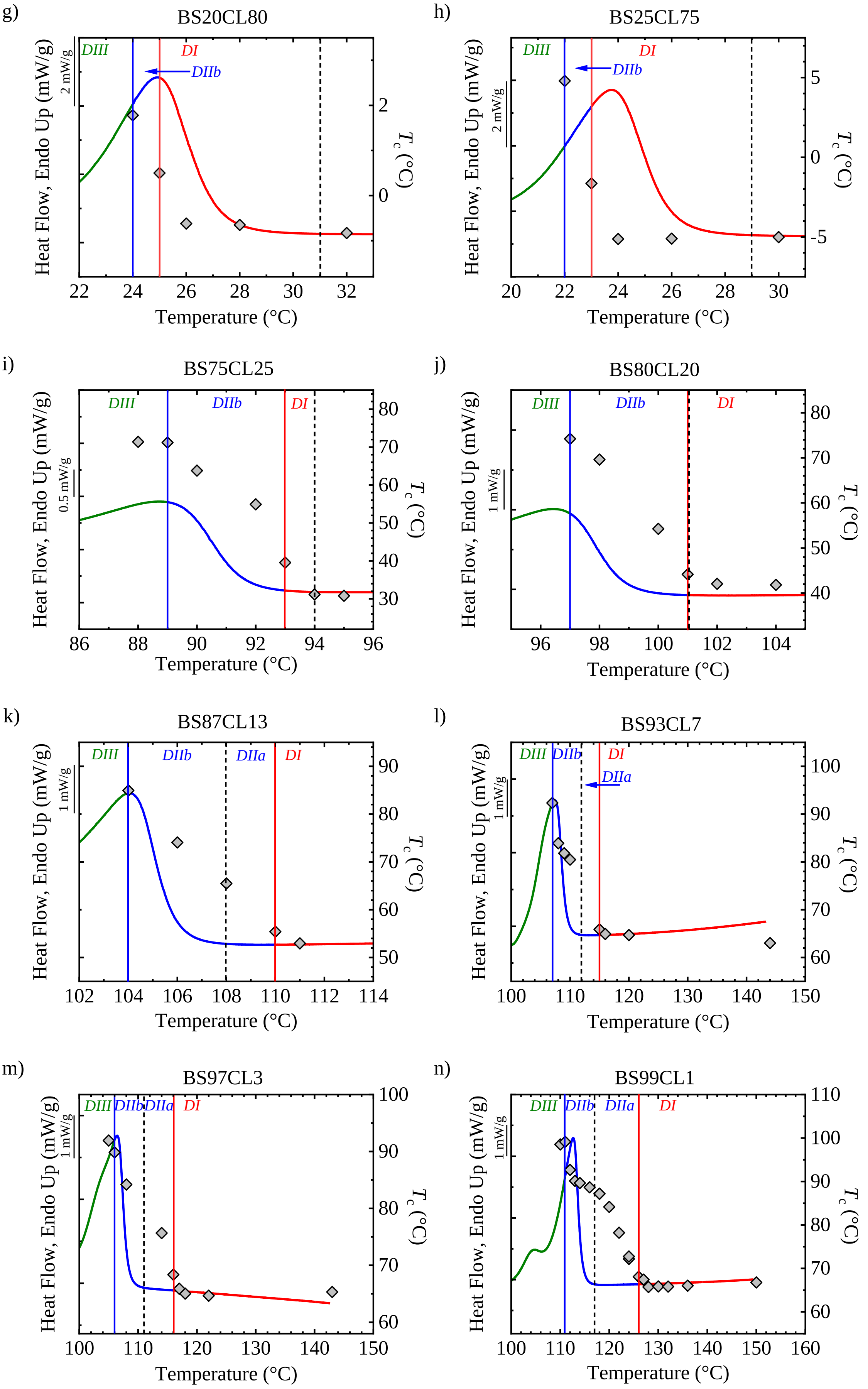
g)
BS20CL80
DIII
DI
DIIb
2 mW/g
Heat Flow, Endo Up (mW/g)
Temperature (°C)
T_c (°C)
h)
BS25CL75
DIII
DI
DIIb
2 mW/g
Heat Flow, Endo Up (mW/g)
Temperature (°C)
T_c (°C)
i)
BS75CL25
DIII
DIIb
DI
0.5 mW/g
Heat Flow, Endo Up (mW/g)
Temperature (°C)
T_c (°C)
j)
BS80CL20
DIII
DIIb
DI
1 mW/g
Heat Flow, Endo Up (mW/g)
Temperature (°C)
T_c (°C)
k)
BS87CL13
DIII
DIIb
DIIa
DI
1 mW/g
Heat Flow, Endo Up (mW/g)
Temperature (°C)
T_c (°C)
l)
BS93CL7
DIII
DIIb
DI
DIIa
1 mW/g
Heat Flow, Endo Up (mW/g)
Temperature (°C)
T_c (°C)
m)
BS97CL3
DIII
DIIb
DIIa
DI
1 mW/g
Heat Flow, Endo Up (mW/g)
Temperature (°C)
T_c (°C)
n)
BS99CL1
DIII
DIIb
DIIa
DI
1 mW/g
Heat Flow, Endo Up (mW/g)
Temperature (°C)
T_c (°C)

o)

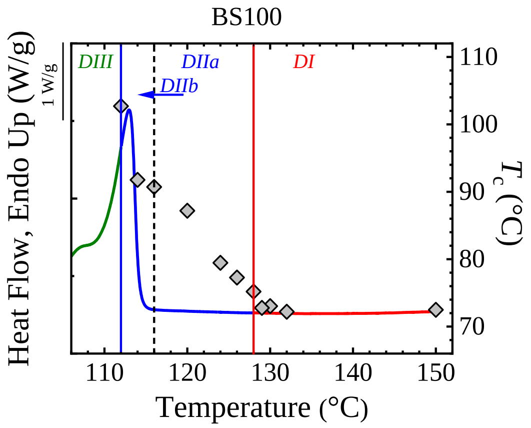

Figure S4. SN results for the copolymers studied in this work. Heating endotherm superimposed to crystallization temperature obtained at several T_s temperatures. Vertical solid lines indicate the transition between domains, whereas the dashed line indicates the end of the melting peak.

References

1. Tessier, M.; Fradet, A. Determination of the degree of randomness in condensation copolymers containing both symmetrical and unsymmetrical monomer units: A theoretical study. *e-Polymers* 2003, 3, 391–406. https://doi.org/10.1515/epoly.2003.3.1.391

2. Fernández-Tena, A.; Pérez-Camargo, R.A.; Coulembier, O.; Sangroniz, L.; Aranburu, N.; Guerrica-Echevarria, G.; Liu, G.; Wang, D.; Cavallo, D.; Müller, A.J. Effect of Molecular Weight on the Crystallization and Melt Memory of Poly (ε-caprolactone) (PCL). Macromolecules 2023, 56, 4602–4620. https://doi.org/10.1021/acs.macromol.3c00234
